# Prognostic factors and risk-stratification model of recurrent or metastatic head and neck squamous cell carcinoma treated with cetuximab containing regimen

**DOI:** 10.1186/s12885-024-12425-0

**Published:** 2024-10-05

**Authors:** Muh-Hwa Yang, Tien-Hua Chen, Hung-Ming Wang, Jason Chia-Hsun Hsieh, Huai-Cheng Huang, Meng-Che Hsieh, Chia-Jui Yen, Shang-Yin Wu, Chun-Hung Hua, Ming-Yu Lien, Yi-Fang Chang, Hui-Ching Wang, Chih-Yen Chien, Tai-Lin Huang, Hsueh-Ju Lu, Jin-Ching Lin, Chen-Chi Wang, Yi-Chun Liu, Jo-Pai Chen, Wei-Chen Lu, Ching-Yi Yiu, Chien-Liang Lin, Pei-Jen Lou, Pen-Yuan Chu

**Affiliations:** 1 Department of Oncology, Division of Medical Oncology, Taipei Veterans General Hospital, Taipei, Taiwan; 2 Department of Internal Medicine, Division of Hematology-Oncology, Chang Gung Memorial Hospital, Linkou, Taoyuan, Taiwan; 3 Department of Internal Medicine, Division of Hematology-Oncology, New Taipei City Municipal TuCheng Hospital, New Taipei City, Taiwan; 4 Department of Oncology, National Taiwan University Hospital and College of Medicine, Taipei, Taiwan; 5 Department of Hematology and Oncology, E-Da Cancer Hospital, Kaohsiung, Taiwan; 6 Department of Oncology, National Cheng Kung University Hospital, Tainan, Taiwan; 7 Department of Otorhinolaryngology, China Medical University Hospital, Taichung, Taiwan; 8 Department of Internal Medicine, Division of Hematology and Oncology, China Medical University Hospital, Taichung, Taiwan; 9 Department of Hematology, MacKay Memorial Hospital, Taipei, Taiwan; 10 Department of Internal Medicine, Division of Hematology and Oncology, Kaohsiung Medical University Hospital, Kaohsiung, Taiwan; 11 Department of Otolaryngology, Kaohsiung Chang Gung Memorial Hospital and Chang Gung University College of Medicine, Kaohsiung City, Taiwan; 12 Department of Hematology-Oncology, Kaohsiung Chang Gung Memorial Hospital, Kaohsiung, Taiwan; 13 Department of Internal Medicine, Division of Hematology and Oncology, Chung Shan Medical University Hospital, Taichung, Taiwan; 14 Department of Radiation Oncology, Changhua Christian Hospital, Changhua, Taiwan; 15 Department of Otolaryngology-Head and Neck Surgery, Taichung Veterans General Hospital, Taichung City, Taiwan; 16 Department of Radiation Oncology, Taichung Veterans General Hospital, Taichung, Taiwan; 17 Department of Oncology, National Taiwan University Hospital Yunlin Branch, Yunlin, Taiwan; 18 Department of Otolaryngology, Chi Mei Medical Center, Liouying, No. 201, Taikang Village, Liouying District, Tainan City, 736402 Taiwan; 19 Department of Internal Medicine, Division of Hematology-Oncology, Chi Mei Medical Center, Liouying, Tainan, Taiwan; 20 Department of Otolaryngology, National Taiwan University Hospital and National Taiwan University College of Medicine, Taipei, Taiwan; 21 Department of Otolaryngology, Taipei Veterans General Hospital, No.201, Sec. 2, Shipai Rd., Beitou District, Taipei City, 11217 Taiwan, ROC

**Keywords:** Cetuximab, Head and neck squamous cell carcinoma, Prognostic factor, Risk-stratification model

## Abstract

**Background:**

In recent years, the addition of cetuximab to chemotherapy has improved treatment outcomes for patients with recurrent/metastatic head and neck squamous cell carcinoma (R/M HNSCC). In this study, we present the real-world survival data of R/M HNSCC patients who received cetuximab-containing regimens from thirteen medical centers in Taiwan, as well as a three-level risk stratification model for this patient population.

**Methods:**

This study enrolled R/M HNSCC patients from thirteen medical centers in Taiwan who received cetuximab-containing regimens from January 1, 2017 to June 6, 2022. The cases were divided into a training cohort and a validation cohort based on the start of treatment. Overall survival (OS) was evaluated in both cohorts and exploratory analysis was performed to identify associated adverse clinical and laboratory factors. The results of the exploratory analysis were used to construct a three-level risk stratification prediction model for OS.

**Results:**

A total of 1434 patients with R/M HNSCC were enrolled in this study and received cetuximab-containing regimens. The overall population had a median OS of 8.57 months (95% CI: 8.07 – 9.08). Multivariate analysis of the training cohort identified poor ECOG performance status, heavy alcohol consumption, and prior adjuvant CCRT or lack of prior RT as adverse prognostic factors. Comparison of laboratory data between patients with OS≦6 and OS > 6 also revealed unfavorable factors, including increased white blood cell count, decreased hemoglobin level, increased platelet count, increased absolute neutrophil count, decreased absolute lymphocyte count, and increased neutrophil-to-lymphocyte ratio. Using forward prediction, a three-level risk stratification prediction model was constructed using the variables of ECOG performance status, alcohol consumption, skin metastasis, modality of radiation therapy, hemoglobin level, and neutrophil-to-lymphocyte ratio. The median OS in the low-risk, intermediate-risk, and high-risk groups were 12.02 months (95% CI 10.44–13.61), 7.5 months (95% CI 7.33–8.17), and 4.01 months (95% CI 3.94–4.08), respectively, with a log-rank test *p*-value < 0.001.

**Conclusion:**

This study presents a three-level risk stratification model with strong prediction ability for OS in R/M HNSCC patients who received cetuximab-containing regimens. The results are based on real-world data and may provide valuable information for clinicians in treatment planning and future drug development.

**Supplementary Information:**

The online version contains supplementary material available at 10.1186/s12885-024-12425-0.

## Introduction

Head and neck squamous cell carcinoma (HNSCC) is a major type of cancer worldwide, with an estimated 750,000 new cases and 360,000 related deaths each year [1]. In the United States, there are 54,000 new HNSCC cases diagnosed and 11,000 related deaths annually, while in Taiwan, HNSCC is the fourth most common type of cancer in men, with a rising incidence rate of 26.44 cases per 100,000 standard population [2, 3]. Despite the abundance of efforts and research aimed at improving the treatment of locally advanced HNSCC through trans-oral or open surgery, radiation therapy (RT), and chemotherapy, the prognosis remains poor and creates a heavy burden on healthcare systems, with a 20–30% recurrence rate in early-stage HNSCC and a 50% recurrence rate in locally advanced cases [4, 5].

For recurrent or metastatic HNSCC (R/M HNSCC) treated with primary concurrent chemoradiation (CCRT) or adjuvant CCRT, treatment options are limited and challenging for physicians [6]. One approach to improve treatment efficacy in these cases is to combine cetuximab with chemotherapy. Studies such as the EXTREME study have shown that a cetuximab-platinum/fluorouracil regimen significantly improves overall survival (OS), progression-free survival, and response rate compared to chemotherapy alone in first-line R/M HNSCC patients [7]. The TPExtreme study also found impressive results, with a median OS of 14.5 months in first-line R/M HNSCC cases when cetuximab was combined with docetaxel and cisplatin [8]. While modest, cetuximab alone is also an option for refractory HNSCC, with a response rate of 13% and a disease control rate of 46% [9]. Our prior retrospective study of cetuximab-containing regimens in R/M HNSCC showed a median OS of 15.6 months in the locoregional recurrence only group [10].

Given these findings, it is crucial to further investigate and identify the clinical factors that impact treatment outcomes, to help physicians evaluate their options. With this in mind, the goal of the current study is to summarize the real-world experiences of thirteen HNSCC medical centers in Taiwan regarding cetuximab-containing regimen treatment outcomes in R/M HNSCC and to recognize and incorporate potential prognostic factors into a three-level risk stratification model.

## Materials and methods

### Study participants and data collection

This study was conducted in accordance with the Declaration of Helsinki and was approved by the Institutional Review Board of thirteen medical centers in Taiwan. A retrospective study was performed on patients who received a cetuximab-containing regimen for R/M HNSCC between January 1st, 2017, and June 6, 2022. The inclusion criteria for participation in the study were as follows: (1) Patients aged 18 or older who had a confirmed diagnosis of HNSCC by pathology, (2) Patients with HNSCC recurrence after curative treatment (surgery, primary CCRT, adjuvant CCRT and RT alone), refractory to induction chemotherapy, or distant metastasis at the time of initial diagnosis, (3) Patients who received documented treatment with a cetuximab-containing regimen for recurrence, refractory, or metastatic disease. Patients who received cetuximab-containing regimen between January 1st, 2017, and December 31, 2019, were included in the training cohort, and patients who received cetuximab-containing regimen between January 1st, 2020, and June 6, 2022, were included in the validation cohort. The patient characteristics collected during the study included: age, gender, Eastern Cooperative Oncology Group (ECOG) performance status, primary site of HNSCC, HPV association, smoking status, alcohol consumption, betel use, clinical staging at initial diagnosis according to the American Joint Committee on Cancer 8th edition (AJCC 8th edition), locoregional recurrence or distant metastasis, site of distant metastasis, primary or secondary disease, prior surgery, modality of radiation therapy, prior induction therapy, and cetuximab-containing regimen.

### Survival outcomes

In this study, OS was the major survival outcome. All the patients in this study came from 13 medical centers and were regularly followed up on a monthly to every three months frequency. Overall survival was defined as from the date beginning the cetuximab-containing regimen to the patient’s last visit date. The data cut-off date was June 17, 2022**.** If no death event reported prior to the data cutoff date, then the overall survival data was censored.

### Covariates of interest

In order to construct risk stratification model for cetuximab-containing treatment in R/M HNSCC, two classes of prognostic factors were applied to covariate analysis: the clinical patient characteristics, and the hematological and biochemistry laboratory data. The clinical characteristics of interest included all the clinical data collected as mentioned above except for AJCC 8th edition clinical staging at initial diagnosis. The hematological and biochemistry laboratory data of interest included: white blood cell count, hemoglobin, platelet count, absolute neutrophil count, absolute lymphocyte count, absolute eosinophil count, NLR (neutrophil-to-lymphocyte ratio), ELR (eosinophil-to-lymphocyte ratio), lactate dehydrogenase, albumin, and blood sugar.

### Risk stratification model construction

To establish the risk stratification model for cetuximab-containing regimen, the risk factors with higher hazard ratio identified by multivariate analysis as mentioned above were assigned a risk score. Risk factors other than a binary variable, such as a continuous variable, were re-grouped into different stratification and examined with multivariate analysis for hazard ratio of OS. Properly stratified risk factors were then assigned risk scores according to the order of stratification. For each patient, the cumulative risk score was the sum of the risk score for each factor in the risk stratification model. The final model included 6 factors: ECOG, alcohol consumption, skin metastasis, modality of RT, hemoglobin level, and NLR, with the cumulative risk score ranged from 0 to 9. To enhance simplicity and establish distinct risk categories, we evenly distributed the total scores into three groups (0–3, 4–6, and 7–9), making it practical for clinical decision-making as a three-level risk stratification groups.

### Statistical analysis

For the analysis of patient characteristics, age was presented as the mean with a 95% confidence interval (95% CI), and the statistical significance between the training and validation cohorts was examined through a two-sample independent t-test. Other categorical variables were presented as numbers and percentages, and the statistical significance between the training and validation cohorts was examined through Pearson's chi-square test.

The OS was analyzed through Kaplan–Meier survival analysis, and the statistical significance between groups was examined through a log-rank test. The impact of clinical characteristics on OS was evaluated through univariate analysis, and variables with a *p*-value less than 0.05 were included in the multivariate analysis.

Hematological and biochemistry lab data in the OS≦6 months and OS > 6 months groups were expressed as the mean with 95% CI, and the significance between groups was examined through a two-sample independent t-test. The clinical characteristic variables that were statistically significant, along with the statistically significant hematological and biochemical variables, were examined through a forward selection procedure by multivariate Cox regression analysis. The selected prognostic variables were designated to risk scores for construction of risk-stratification model, as mentioned above. The OS of the three risk groups was then examined through Kaplan–Meier analysis and log-rank test in the validation cohort. The statistical analyses were performed using SPSS v22.0.

## Results

### The clinical characteristics of the whole, training, and validation cohorts

From January 1st, 2017 to June 6, 2022, a total of 1,434 patients who had received cetuximab-based treatment for R/M HNSCC were enrolled in the analysis. The training cohort consisted of 1,157 patients enrolled from January 1st, 2017 to December 31, 2019, while the validation cohort consisted of 277 patients enrolled from January 1st, 2020 to June 6, 2022. Table 1 presents the patient characteristics of the training and validation cohorts. There were no significant differences between the two cohorts in regards to age, gender, smoking status, alcohol consumption, betel use, staging at initial diagnosis, locoregional recurrence or distant metastasis, prior surgery, and modality of RT. However, there were more cases with good performance (ECOG = 0) and oropharynx cancer, as well as fewer HPV-associated oropharynx cancer cases, cases with liver metastasis, and cases who had received prior induction therapy in the validation cohort, compared to the training cohort.

**Table 1:** Patient characteristics of training cohort and validation cohort

	**Training cohort (*N* = 1157)**		**Validation cohort (*N* = 277)**		***p*-Value**
**Age**, years (mean, 95%CI)^a^	57.2	56.7—57.7	56.5	55.3–57.7	0.320
					0.420
< 65	913	78.9%	225	81.2%	
≥ 65	242	20.9%	52	18.8%	
**Gender**					0.282
Male	1078	93.2%	263	94.9%	
Female	79	6.8%	14	5.1%	
**ECOG performance status**^a^					** < 0.001**
0	125	10.8%	73	26.4%	
1	882	76.2%	183	66.1%	
≥ 2	148	12.8%	20	7.2%	
**Primary site**					**0.003**
Oral cavity	615	51.9%	123	46.1%	
Oropharynx	215	19.6%	74	26.1%	
Hypopharynx	207	18.7%	62	20.0%	
Larynx	74	6.1%	13	6.1%	
Others	46	3.7%	5	1.7%	
**HPV-associated oropharynx cancer**	49	22.7%	12	16.2%	** < 0.001**
**Smoking status**^**a**^					0.846
Never	173	13.1%	43	15.5%	
Current or former	976	74.0%	234	84.5%	
**Alcohol consumption**^**a**^					0.325
Abstinent or occasional	777	58.9%	211	76.2%	
Heavy drinker	239	18.1%	55	19.9%	
**Betel use**^**a**^					0.223
Never	330	28.5%	92	33.2%	
Current or former	782	67.6%	183	66.1%	
**Staging at initial diagnosis**^**a**^ (AJCC 8th)					0.240
0	2	0.2%	0	0.0%	
I	66	5.7%	24	8.7%	
II	72	6.2%	28	10.1%	
III	83	7.2%	25	9.0%	
IVA	343	29.6%	85	30.7%	
IVB	181	15.6%	68	24.5%	
IVC	71	6.1%	18	6.5%	
**Locoregional recurrence/ distant metastasis**^**a**^					0.064
Locoregional recurrence only	476	41.1%	107	38.6%	
Distant metastatic with/ without locoregional disease	557	48.1%	162	58.5%	
**Site of distant metastasis**^**b**^					
Lung	303	26.2%	81	29.2%	0.271
Distant lymph node	133	11.5%	37	13.4%	0.366
Bone	137	11.8%	22	7.9%	0.068
Liver	57	4.9%	6	2.2%	**0.046**
Skin	78	6.7%	11	4.0%	0.091
Brain	12	1.0%	4	1.4%	0.240
**Primary or secondary disease**^**a**^					** < 0.001**
Primary disease	947	81.8%	263	94.9%	
Second primary malignancy	203	17.5%	12	4.3%	
**Prior Surgery**^**a**^					0.104
No	487	42.1%	104	37.5%	
Yes	640	55.3%	171	61.7%	
**Modality of RT**^**a**^					0.086
Primary CCRT	478	41.3%	116	41.9%	
RT alone	30	2.6%	4	1.4%	
Adjuvant CCRT	222	19.2%	41	14.8%	
No RT	368	31.8%	107	38.6%	
**Prior induction therapy**^**a**^					** < 0.001**
Induction therapy	351	30.3%	55	19.9%	
No induction therapy	747	64.6%	213	76.9%	
**Cetuximab-based regimen**^**a**^					** < 0.001**
Cetuximab + PF	344	29.7%	163	58.8%	
Cetuximab + non-PF	809	69.9%	112	40.4%	

*ECOG* Eastern Cooperative Oncology Group, *HPV* Human papillomavirus, *AJCC* American Joint Committee on Cancer, *RT* radiation therapy, *PF* platinum-fluorouracil

Statistical significant *p*-value is expressed in bold

^a^Missing data: Age: 2; ECOG: 3; Smoking status: 8; Alcohol consumption: 152; Betel chewer: 47; Staging at initial diagnosis: 368; Extent of recurrent/ refractory disease: 132; Primary or secondary disease: 9. Prior surgery: 32; Modality of RT: 68; Prior induction therapy: 68; Cetuximab-based regimen: 6

^b^There may be more than one metastasis site in a patient

### The overall survival of whole, training, and validation cohort

**Fig. 1:**
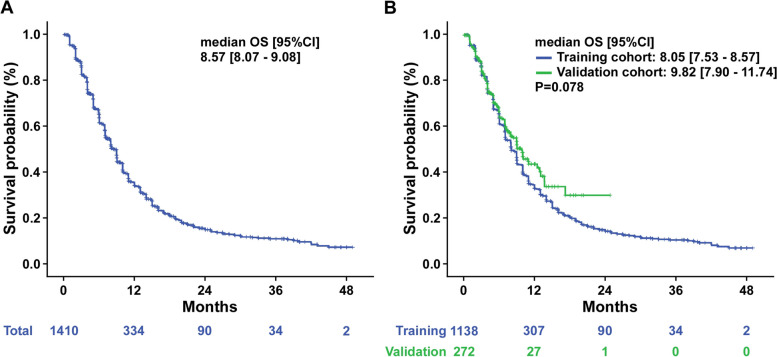
Kaplan–Meier plot for overall survival (OS). **A** OS for the whole cohort. **B** OS for the training cohort and validation cohort

The median follow-up time, median OS, 1-year and 2-year estimated survival rates for the entire cohort (including both the training and validation cohorts), the training cohort, and the validation cohort are presented in Table 2. At the data cutoff date, totally 1034 death events were reported, and 21 participants were lost of follow up. The rate of lost follow up was 1.46%. For the entire cohort, the median follow-up time was 20.96 months (95% CI: 18.93—23.00), the median OS was 8.57 months (95% CI: 8.07—9.08), and the 1-year and 2-year estimated survival rates were 32.2% and 14.7% respectively **(**Fig. 1**)**. The median follow-up time for the training cohort was 26.97 months (95% CI: 25.01—28.94), the median OS was 8.05 months (95% CI: 7.53—8.57), and the 1-year and 2-year estimated survival rates were 31.2% and 14.1% respectively. The median follow-up time for the validation cohort was 8.94 months (95% CI: 8.07—9.81), the median OS was 9.82 months (95% CI: 7.90—11.74), and the 1-year and 2-year estimated survival rates were 40.0% and 30.1% respectively. There was a non-significant increase in the median OS in the validation cohort compared to the training cohort (*p* = 0.078).

**Table 2:** Overall survival of total cohort, training cohort, and validation cohort

	Median OS (months)	95%CI	*P*-value	1-year estimated survival rate	2-year estimated survival rate	Median follow up time (months)	95%CI
Total cohort	8.57	8.07—9.08		32.2%	14.7%	20.96	18.93—23.00
			0.078				
Training cohort	8.05	7.53—8.57		31.2%	14.1%	26.97	25.01—28.94
Validation cohort	9.82	7.90—11.74		40.0%	30.1%	8.94	8.07—9.81

*OS* overall survival, *CI* confidence interval

### Univariate and multivariate analysis of clinical characteristics on OS from training cohort

The results of the univariate and multivariate analysis of the clinical characteristics affecting OS from the training cohort are presented in Table 3. The univariate analysis reveals several unfavorable clinical features for patients with recurrent or metastatic R/M HNSCC treated with cetuximab regimen, including poor ECOG performance status, smoking history, heavy alcohol consumption, larynx primary tumor, skin metastasis, and lack of prior adjuvant CCRT or absence of prior RT. Upon further examination through multivariate analysis, it was determined that poor ECOG performance status, heavy alcohol consumption, and lack of prior adjuvant CCRT or absence of prior radiation therapy were still negatively associated with the outcomes of R/M HNSCC treated with cetuximab regimen.

**Table 3:** Univariate and multivariate analysis of the training cohort

	**Univariate analysis**			**Multivariate analysis**		
	HR	95% CI	*p*-value	HR	95% CI	*p*-value
**Age (years)**						
< 65	ref					
≥ 65	0.871	0.737—1.029	0.104			
**Gender**						
Female	ref					
Male	1.04	0.800—1.351	0.771			
**ECOG**						
0	ref			ref		
1	1.391	1.116—1.733	**0.003**	1.435	1.131—1.821	**0.003**
≥ 2	2.695	2.058—3.529	** < 0.001**	2.960	2.207—3.969	** < 0.001**
**Smoking status**						
Never	ref			ref		
Current or former	1.346	1.108—1.635	**0.003**	1.185	0.955—1.469	0.122
**Betel Chewer**						
Never	ref					
Current or former	1.105	0.953—1.282	0.185			
**Alcohol consumption**						
Abstinent or occasional	ref			ref		
Heavy drinker	1.368	1.167—1.603	** < 0.001**	1.329	1.123—1.574	**0.001**
**Extent of recurrent/ refractory disease**						
Locoregional recurrence only	ref					
Distant metastatic with/ without locoregional disease	1.132	0.984—1.302	0.083			
**Primary site**						
Oral cavity	ref			ref		
Oropharynx	0.934	0.783—1.113	0.443	0.930	0.759—1.141	0.488
Hypopharynx	0.839	0.702—1.003	0.054	0.939	0.763—1.156	0.552
Larynx	0.695	0.520—0.930	**0.014**	0.757	0.556—1.03	0.077
**Site of distant metastasis**						
Lung	1.071	0.921—1.246	0.374			
Distant lymph node	0.872	0.704—1.081	0.213			
Bone	1.218	0.998—1.486	0.052			
Liver	1.104	0.813—1.500	0.526			
Skin	1.404	1.094—1.801	**0.008**	1.306	0.996—1.712	0.053
Brain	1.43	0.809—2.528	0.219			
**Prior Surgery**						
No	ref					
Yes	1.095	0.956—1.254	0.189			
**Modality of RT**						
RT alone	ref			ref		
Primary CCRT	1.451	0.903—2.331	0.124	1.323	0.81—2.162	0.263
Adjuvant CCRT	1.946	1.200—3.155	**0.007**	1.685	1.018—2.791	**0.043**
No RT	1.766	1.097—2.842	**0.019**	1.466	0.893—2.406	**0.130**
**Prior induction therapy**						
Induction therapy	ref					
No induction therapy	1.106	0.953—1.283	0.185			

*ECOG* Eastern Cooperative Oncology Group, *HPV* Human papillomavirus, *AJCC* American Joint Committee on Cancer, *RT* radiation therapy

Statistical significant *p*-value is expressed in bold

### Hemogram and biochemistry analysis in groups with OS less than or more than 6 months

The analysis of hemogram and biochemistry data was performed between two groups, those with OS less than or equal to 6 (OS≦6) and those with OS greater than 6 (OS > 6), as presented in Table 4. Several hemogram and biochemistry parameters were found to be significantly different between the training cohort and validation cohort, including white blood cell count (10,088 × 10^9^/L vs. 7773 × 10^9^/L, *p* < 0.001), hemoglobin (10.69 g/dL vs. 11.57 g/dL, *p* < 0.001), platelet count (30.46 × 10^4^/L vs. 28.09 × 10^4^/L, *p* = 0.002), absolute neutrophil count (8475 × 10^9^/L vs. 5976 × 10^9^/L, *p* < 0.001), absolute lymphocyte count (868 × 10^9^/L vs. 1060 × 10^9^/L, *p* < 0.001), and the neutrophil-to-lymphocyte ratio (NLR) (14.61 vs. 7.59, *p* < 0.001). No significant differences were observed between the OS≦6 and OS > 6 groups with respect to the absolute eosinophil count, the eosinophil-to-lymphocyte ratio (ELR), lactate dehydrogenase, albumin, or blood sugar levels. Although not statistically significant, a trend of elevated ELR (0.36 vs. 0.24, *p* = 0.088) and lactate dehydrogenase (198.08 U/L vs. 179.09 U/L, *p* = 0.061) was observed in the OS≦6 group.

**Table 4:** Hemogram and Biochemistry study in OS≦ 6 months and in OS > 6 months groups

	≦ 6 months (*N* = 588)			> 6 months (*N* = 826)			*p*-value
	N	Mean	SD	N	Mean	SD	
White blood cell (× 10^9^/L)	587	10,088.86	7564.57	823	7773.71	6160.31	** < 0.001**
Hemoglobin (g/dL)	585	10.69	2.12	823	11.57	1.94	** < 0.001**
Platelet (× 10^4^/L)	583	30.46	14.78	822	28.09	12.64	**0.002**
Absolute neutrophil count (× 10^9^/L)	556	8475.86	7390.23	773	5976.75	5739.98	** < 0.001**
Absolute lymphocyte count (× 10^9^/L)	578	868.88	734.24	817	1060.34	717.8	** < 0.001**
Absolute eosinophil count (× 10^9^/L)	513	173.45	260.82	779	169.15	181.39	0.745
NLR (neutrophil-to-lymphocyte ratio)	555	14.61	19.01	773	7.59	8.78	** < 0.001**
ELR (eosinophil-to-lymphocyte ratio)	513	0.36	2.88	779	0.19	0.24	0.088
Lactate dehydrogenase (U/L)	116	198.08	106.74	195	179.09	70.78	0.061
Albumin (g/dL)	329	4.15	9.13	445	3.98	0.47	0.697
Blood sugar (mg/dL)	123	123.18	39.86	222	120.95	47.89	0.662

Statistical significant *p*-value is represented in bold

### Risk stratification model of R/M HNSCC treated with cetuximab containing regimen

**Fig. 2:**
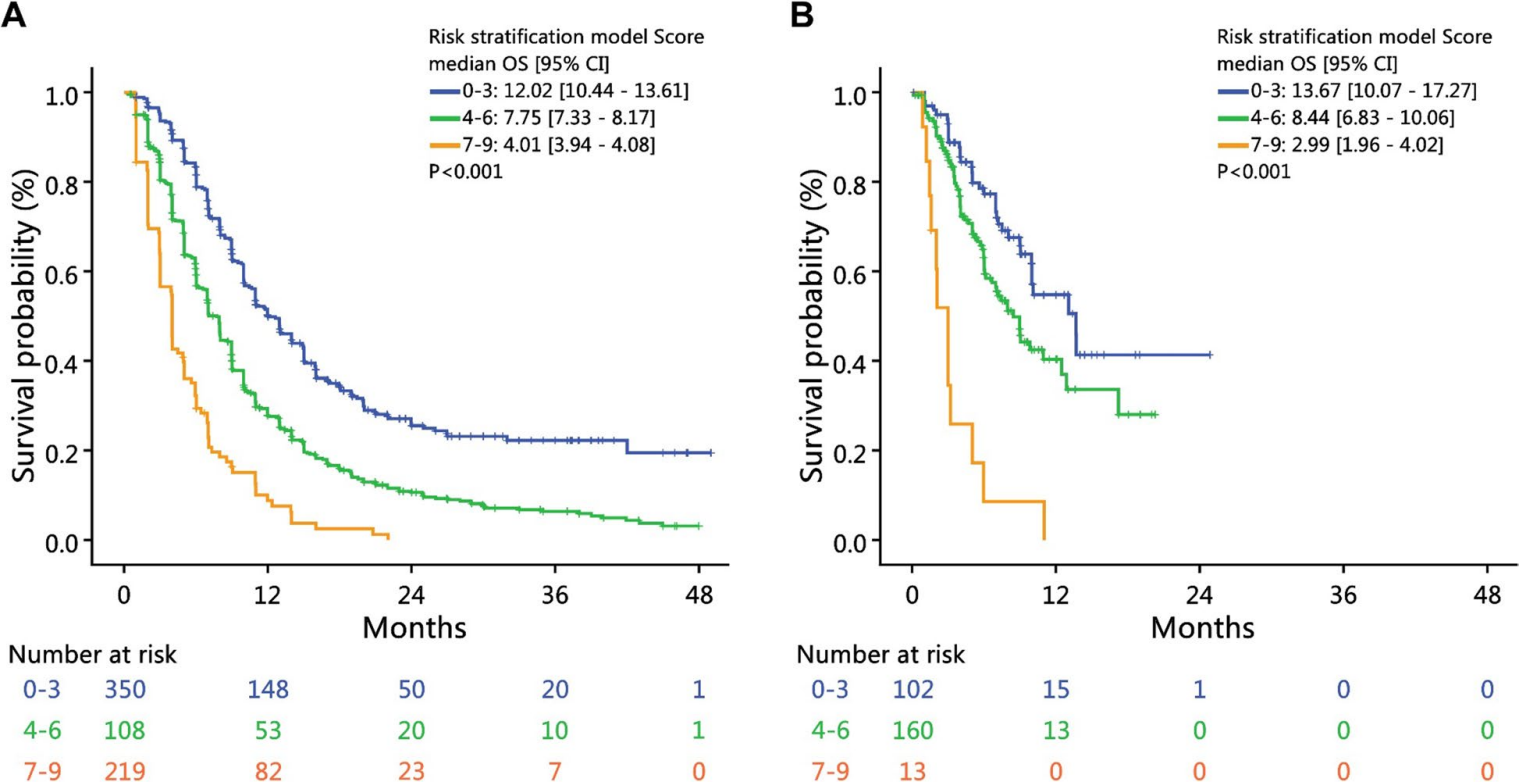
Kaplan–Meier plot for overall survival (OS) according to risk stratification. **A** OS according to risk stratification in training cohort. **B** OS according to risk stratification in the validation cohort

A risk stratification model for R/M HNSCC treated with cetuximab-containing regimens was developed based on the results of univariate and multivariate analysis of clinical and laboratory variables. The forward selection method was employed, resulting in the inclusion of the following parameters: ECOG score (0, 1, or ≧2), alcohol consumption, presence of skin metastasis, type of radiation therapy, hemoglobin, and NLR. The details of these risk stratification factors are presented in Table 5.. The risk groups were then defined as low risk (score of 0–3), intermediate risk (score of 4–6), and high risk (score of 7–9) (Table 6). In the training cohort, the median OS was 12.02 months (95% CI 10.44–13.61), 7.75 months (95% CI 7.33–8.17), and 4.01 months (95%CI 3.94–4.08) for the low risk, intermediate risk, and high risk groups, respectively. The log-rank test showed significant differences (*p* < 0.001) between the different risk groups. The risk stratification model was then applied to the validation cohort and the median OS was found to be 13.67 months (95% CI 10.07–17.27), 8.44 months (95% CI 6.83–10.06), and 2.99 months (95% CI 1.96–4.02) for the low risk, intermediate risk, and high risk groups, respectively. The log-rank test also showed significant differences (*p* < 0.001) between the different risk groups in the validation cohort. The Kaplan–Meier survival curves for the three risk groups in the training and validation cohorts are shown in Fig. 2. Furthermore, we applied the risk stratification model to the overall cohort, and the median OS was found to be 12.65 months (95% CI 11.18—14.12), 7.95 months (95% CI 7.41—8.50), and 4.01 months (95% CI 3.49 – 4.52) in the low risk, intermediate risk and high risk groups. The log-rank test showed significant differences (*p* < 0.001) for all three comparison of each two of risk groups (Supplementary Table 1).

**Table 5.** Multivariate analysis of the risk stratification variables from training cohort

	HR	95% CI	*p*-value	Given point
**ECOG performance status**				
0	ref			0
1	1.333	1.048—1.695	**0.019**	1
≥ 2	2.438	1.802—3.298	** < 0.001**	2
**Alcohol consumption**				
Abstinent or occasional	ref			0
Heavy drinker	1.366	1.156—1.615	**0.001**	1
**Site of distant metastasis**				
Skin	1.366	1.032—1.808	**0.029**	1
**Modality of RT**				
RT alone	ref			0
Primary CCRT	1.301	0.772—2.192	0.323	1
Adjuvant CCRT	1.701	0.999—2.898	0.051	1
No RT	1.558	0.920—2.638	0.099	1
**Hemoglobin (g/dL)**				
≥ 11	ref			0
< 11	1.423	1.218—1.662	** < 0.001**	1
**NLR**				
< 2.5	ref			0
2.5 ~ < 5	1.392	1.039—1.866	**0.027**	1
5 ~ < 10	2.036	1.529—2.712	** < 0.001**	2
≥ 10	2.620	1.950—3.521	** < 0.001**	3

*ECOG* Eastern Cooperative Oncology Group, *RT* radiation therapy, *NLR* neutrophil-to-lymphocyte ratio

*P*-value with statistical significance is in bold

**Table 6:** Overall survival of three risk group in training cohort and in validation cohort

Risk stratification model Score		Training cohort				Validation cohort		
	N	Median (months)	95% CI	*P*-value	N	Median (months)	95% CI	*P*-value
				** < 0.001**				** < 0.001**
0–3	350	12.02	10.44—13.61		102	13.67	10.07—17.27	
4–6	680	7.75	7.33—8.17		160	8.44	6.83—10.06	
7–9	109	4.01	3.94—4.08		13	2.99	1.96—4.02	

18 patients in the training cohort and 2 patients in the validation cohort had missing data in the risk stratification factors and was not included in this analysis

*CI* confidence interval

## Discussion

In this study, we have revealed the real-world median OS of 8.57 months for R/M HNSCC patients treated with a cetuximab-containing regimen. Additionally, we have identified multiple adverse clinical features in this real-world cohort, including poor ECOG performance status, heavy alcohol use, prior adjuvant CCRT or lack of prior RT. Our study also disclosed multiple hematological parameters associated with unfavorable OS outcomes in the cetuximab-containing regimen-treated R/M HNSCC population, including an increased white blood cell count, decreased hemoglobin level, increased platelet count, increased absolute neutrophil count, decreased absolute lymphocyte count, and increased NLR. To aid physicians in predicting the treatment outcomes of cetuximab-containing regimens for R/M HNSCC, we also presented a three-level risk stratification model (low-risk, intermediate-risk, and high-risk).

Previous studies have revealed adverse clinical features for R/M HNSCC populations. One study retrospectively evaluating 482 R/M HNSCC patients in a single institution revealed that older age and advanced tumor stage were unfavorable risk factors for OS [11]. Another study analyzing patients in the E1393 and E1395 phase III clinical trials who received platinum/fluorouracil treatment for R/M HNSCC revealed that weight loss exceeding 5%, poor ECOG performance, non-oropharynx SCC, prior RT, and well to moderate tumor cell differentiation were unfavorable risk factors for OS [12]. Another study retrospectively evaluated cetuximab plus platinum-based chemotherapy in the first-line treatment of R/M HNSCC in six Italian medical centers and showed that poor ECOG performance, platinum resistance, and residual tumor at primary sites were unfavorable risk factors for OS [13]. Our study differs from these prior studies mainly in terms of the enrolled population and the clinical data types collected. All of the cases in our study received cetuximab-containing treatment, while most of these prior studies did not focus specifically on cetuximab treatment efficacy. Some data types were also not collected in our database, such as the magnitude of weight loss or tumor cell differentiation. Nonetheless, our study showed agreement with prior studies that poor ECOG performance is an independent adverse factor for OS in this patient population. Furthermore, we also disclosed heavy alcohol use, prior adjuvant CCRT, or lack of prior RT as newly discovered independent adverse factors for OS.

In addition to these clinical features, we also collected hematological and biochemical laboratory data for analysis of the difference between OS ≤ 6 and OS ≥ 6 groups. Prior studies have indicated that the NLR, platelet-lymphocyte ratio, and lymphocyte-to-monocyte ratio are factors associated with poor prognosis in HNSCC [14–17]. Anemia has also been considered an adverse effect on both HNSCC patient survival and RT efficacy, potentially due to increased tumor hypoxia caused by the anemia, leading to decreased sensitivity to RT [18, 19]. Our prior study also showed that tumor-associated eosinophilia is associated with inferior OS, increased tumor migration ability, and elevated angiogenesis in tumor tissue [20]. Our study analysis agreed with many of the aforementioned unfavorable risk factors, including increased NLR, decreased hemoglobin level, and a non-significantly increased ELR.

In the aforementioned study, a two-level survival model was established by analyzing the adverse risk factors present in patients who underwent platinum/fluorouracil treatment as part of phase III clinical trials E1393 and E1395 for recurrent/metastatic head and neck squamous cell carcinoma (R/M HNSCC) [12]. The survival curves were clearly differentiated between low-risk and high-risk groups, with a median OS of 12 months and 6 months, respectively. A subsequent study validated the two-level risk stratification model and demonstrated accurate predictions between low-risk and high-risk groups (median OS of 14 months versus 10 months, *p*= 0.03) [21]. In our study, a three-level risk stratification model was developed by incorporating statistically significant clinical and hematological factors, which also showed strong prediction ability among the three groups (median OS of 12.02, 7.75, and 4.01 months, *p* < 0.001). This risk-stratification model provides valuable insight for clinicians to make informed treatment plans. As patients undergo evaluation, their risk scores, based on identified factors such as ECOG performance status, alcohol use, prior treatments, and hematological parameters, are calculated and used to categorize them into low, intermediate, or high-risk groups. In the clinical setting, this model becomes a valuable tool for risk assessment prior to cetuximab-containing treatment. For patients in the high-risk group, limited treatment efficacy under cetuximab-containing regimen can be expected. Therefore, incorporation of immunotherapy or enrollment into clinical trials should be discussed earlier in the course with the patients.

There are several limitations to this study, given its retrospective and uncontrolled nature. Firstly, there is heterogeneity in the chemotherapy partner of cetuximab used across all 13 medical centers, which may pose challenges in further analysis. Secondly, the units used for documenting biochemical data may vary between centers, leading to data loss in some types of information, such as the CRP level in our study population. Despite these limitations, this study still offers a comprehensive view of the real-world experience of cetuximab-containing regimens for R/M HNSCC in a large study population.

## Conclusion

This study presents a three-level risk stratification model with strong prediction ability for OS in R/M HNSCC patients who received cetuximab-containing regimens. The results are based on real-world data and may provide valuable information for clinicians in treatment planning and future drug development.

## Supplementary Information

Supplementary Material 1.

## Data Availability

The data used and analyzed in this study are available from the corresponding author upon reasonable request.
