# Supplementary material for: Prognostic factors and risk-stratification model of recurrent or metastatic head and neck squamous cell carcinoma treated with cetuximab containing regimen
Source: BMC Cancer. 2024 Oct 5;24:1227. doi: 10.1186/s12885-024-12425-0 (PMC11452986; doi:10.1186/s12885-024-12425-0)

**Supplementary Table 1. Overall survival of three risk group in overall cohort**

| Risk stratification  model |  |  | All (N=1414) | | |  |  |  |
| --- | --- | --- | --- | --- | --- | --- | --- | --- |
|  |  | N | Median  (months) | 95% CI | P-value |  |  | P-value |
|  |  |  |  |  | **<0.001** |  |  |  |
| Score: 0-3 |  | 452 | 12.65 | 11.18 - 14.12 |  |  | 0-3 vs. 4-6 | **<0.001** |
| Score: 4-6 |  | 840 | 7.95 | 7.41 - 8.50 |  |  | 4-6 vs. 7-9 | **<0.001** |
| Score: 7-9 |  | 122 | 4.01 | 3.49 - 4.52 |  |  | 0-3 vs. 7-9 | **<0.001** |


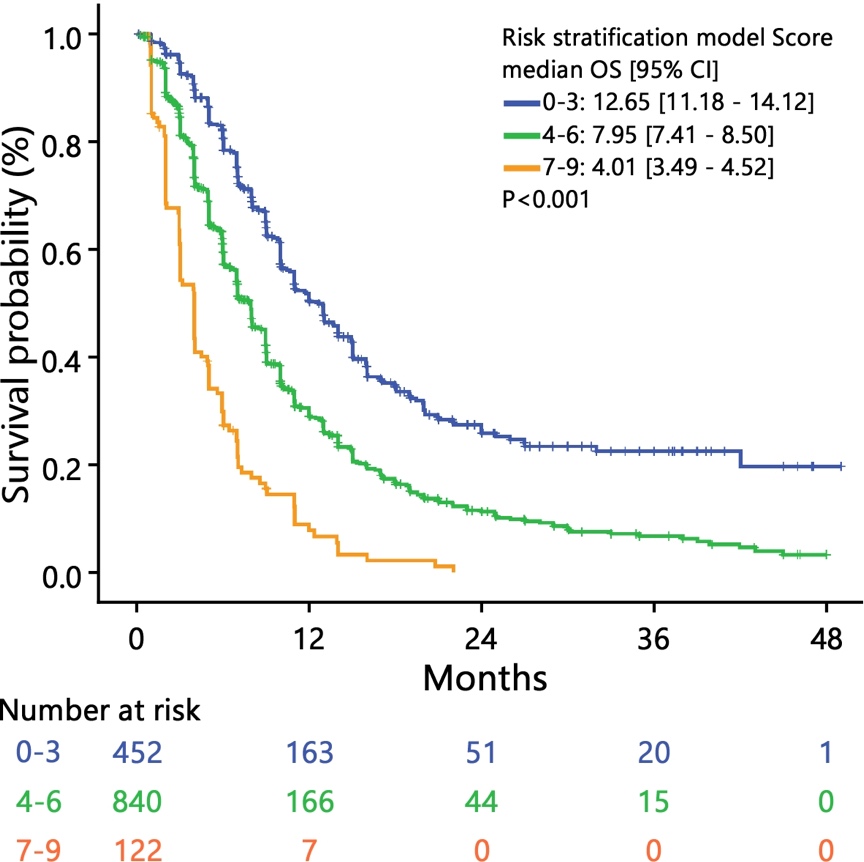

Supplement: Supplementary file 1 — Supplementary Material 1. [file 12885_2024_12425_MOESM1_ESM.docx]
